# Complement factor B is essential for the proper function of the peripheral auditory system

**DOI:** 10.3389/fneur.2023.1214408

**Published:** 2023-07-25

**Authors:** LaShardai N. Brown, Jeremy L. Barth, Shabih Jafri, Jeffrey A. Rumschlag, Tyreek R. Jenkins, Carl Atkinson, Hainan Lang

**Affiliations:** ^1^Department of Pathology and Laboratory Medicine, Medical University of South Carolina, Charleston, SC, United States; ^2^Department of Regenerative Medicine and Cell Biology, Medical University of South Carolina, Charleston, SC, United States; ^3^Division of Pulmonary Medicine, University of Florida, Gainesville, FL, United States

**Keywords:** cochlea, complement factor B, macrophage, auditory nerve, glial cell, myelination, stria vascularis, hearing loss

## Abstract

Sensorineural hearing loss is associated with dysfunction of cochlear cells. Although immune cells play a critical role in maintaining the inner ear microenvironment, the precise immune-related molecular mechanisms underlying the pathophysiology of hearing loss remain unclear. The complement cascade contributes to the regulation of immune cell activity. Additionally, activation of the complement cascade can lead to the cellular opsonization of cells and pathogens, resulting in their engulfment and elimination by phagocytes. Complement factor B (fB) is an essential activator protein in the alternative complement pathway, and variations in the fB gene are associated with age-related macular degeneration. Here we show that mice of both sexes deficient in fB functional alleles (fB^−/−^) demonstrate progressive hearing impairment. Transcriptomic analysis of auditory nerves from adult mice detected 706 genes that were significantly differentially expressed between fB^−/−^ and wild-type control animals, including genes related to the extracellular matrix and neural development processes. Additionally, a subset of differentially expressed genes was related to myelin function and neural crest development. Histological and immunohistochemical investigations revealed pathological alterations in auditory nerve myelin sheathes of fB^−/−^ mice. Pathological alterations were also seen in the stria vascularis of the cochlear lateral wall in these mice. Our results implicate fB as an integral regulator of myelin maintenance and stria vascularis integrity, underscoring the importance of understanding the involvement of immune signaling pathways in sensorineural hearing loss.

## 1. Introduction

Sensorineural hearing loss (SNHL) is a multifactorial disorder resulting from dysfunction of cochlear structures. Despite the growing prevalence of SNHL worldwide (1), the pathological mechanisms underlying SNHL remain elusive. Recent findings highlight the important role of inflammation, pro-inflammatory mediators, and dysregulation of the immune system in exacerbating cellular degeneration in neurological disorders including age-related hearing loss (2–4). Dysregulation of the complement signaling pathways may contribute to age-related pathological alterations in the brain and neurological disorders (5). The complement pathway has been associated with several cellular processes in the central nervous system, including synaptic plasticity and synaptic pruning during development (6, 7), axonal pruning [e.g., via microglial trogocytosis; (8)], and blood-brain barrier mediation (9). Complement signaling has also been found to contribute to neurodegenerative pathologies, including multiple sclerosis (10), drug-induced excitotoxicity (11), and Alzheimer’s Disease (12). However, the impacts of the different complement pathways in healthy and impaired auditory systems are just beginning to be elucidated.

Complement signaling is comprised of over 50 fluid-phase and membrane-bound proteins that are important for activating the innate immune system. The complement cascade can be activated by 3 pathways: classical, lectin, and alternative pathways (13, 14). Recent studies have highlighted the potential role of classical complement signaling in auditory function. For instance, *C1ql1* is expressed in the outer hair cells of young adult mice (15) and global *C1ql1-*deficient mice show a decline in hearing sensitivity and pathological alterations in the auditory nerve and outer hair cells (15, 16). Additionally, levels of complement factor I (CFI, known as C3b/C4b inactivator) were increased in the sensory epithelium following noise trauma in a rodent model (17). These observations reveal a potential role for complement signaling in regulating the development and maintenance of cochlear structure and function.

Interestingly, the alternative pathway amplifies the activation of the classical and lectin pathways through the activity of complement factor B (fB). During spontaneous activation of the alternative pathway, fB binds to and cleaves C3 to generate C3b. Fragments of fB bind to C3b to catalyze the creation of the alternative pathway C3 convertase, C3bBb, which then amplifies complement activation and contributes to the formation of the membrane attack complex (13, 18). A previous study reported that fB was highly expressed in the middle ear epithelium following *Streptococcus pneumoniae* infection in rodent models of otitis media, demonstrating that alternative complement activation was essential for protection against otitis media (19). Here, we examined the role of alternative complement protein fB in the peripheral auditory system using a well-characterized mouse model of fB deficiency (fB^−/−^) (20–22). Using a combination of auditory brainstem response (ABR) measurements, RNA-sequencing, electron microscopy ultrastructural analyses, and quantitative immunohistochemical investigations, our data revealed an important role for alternative complement signaling in the peripheral auditory system. We found that fB deficiency results in reduced hearing sensitivity and declines in auditory nerve function, along with significant alterations of auditory nerve myelin and defects in the interdigitation of cells in the stria vascularis of the cochlear lateral wall. These results suggest that the alternative complement pathway plays a role in regulating proper auditory function and the development and maintenance of the cochlear structure.

## 2. Materials and methods

### 2.1. Animals

CBA/CaJ and C57Bl/6J mice are established models for hearing research. Since C57BL/6J mice maintain an *Ahl* gene mutation and exhibit early-onset age-related morphological alterations (23), CBA/CaJ mice were used for the investigation of RNA profiling to explore complement-related expression in the developing cochlea. For analysis of complement-related gene expression in the developing cochlea, P3, P7, P14, and P21 CBA/CaJ mice of both sexes were used. CBA/CaJ mice, originally purchased from the Jackson Laboratory (Bar Harbor, ME), were bred in-house in a low-noise environment at the Animal Research Facility of the Medical University of South Carolina (MUSC; Charleston, SC, United States). Factor B deficient (fB^−/−^) mice were established previously (20) and provided by Dr. Carl Atkinson. The model was generated by targeted homologous recombination in embryonic stem cells, resulting in mice with non-functional fB alleles. These mice show no gross difference in phenotype when compared to wild-type (WT, C57BL/6 J) mice. Young adult (1.5–4 months) WT and fB^−/−^ mice of both sexes were used in this study. All aspects of animal research were conducted in accordance with the guidelines of the Institutional Animal Care and Use Committee of MUSC (IACUC-2020-01132). All mice received food and water *ad libitum* and were maintained on a 12-h light/dark cycle.

### 2.2. Assessment of auditory functions

Auditory brainstem responses (ABR) were measured as previously described (24–26). Briefly, mice were anesthetized by intraperitoneal injection of xylazine (20 mg/kg) and ketamine (100 mg/kg) and placed in a sound-isolation room. Depth of anesthesia was regularly examined by lack of foot-pinch response. Using Tucker-Davis Technologies System III equipment (Tucker-Davis Technologies, Gainesville, FL, United States) and a SigGen software package (Version 4.4.1), acoustic stimuli were generated, and sound calibration was completed using an IPC Microphone System (PCB Piezotronics Inc., Depew, NY, United States). The acoustic stimuli were presented to the animal’s ear canal through a 3–5 mm outer diameter plastic tube. Auditory function was assessed by evoking responses at half-octave frequencies from 4 kHz to 45.2 kHz with 5 ms duration tone pips, with 0.5 ms cos^2^ rise/fall times, delivered at a rate of 31 times/s. Sound levels were reduced from 90 to 5 dB sound pressure level (SPL) in 5 dB SPL intervals. Hearing thresholds were identified as the lowest intensity that produced a reproducible response wave.

### 2.3. Immunohistochemistry and quantitative analysis

Cochleae were collected and fixed as described previously (24). Tissues were then embedded in Tissue-Tek OCT Compound (VWR, Radnor, PA, United States) and sectioned at 10 μm thickness. Tissues were washed with 10 mM PBS and permeabilized with 2% bovine serum albumin and 0.4% Triton X-100 (Sigma, Burlington, MA, United States) for 15 min. Sections were then blocked with normal goat serum solution for 1 h at room temperature and incubated overnight with primary antibodies at 4°C: rabbit anti-Iba1 [Wako Chemicals USA Inc., Richmond, VA, United States (1:250)], mouse anti-Neurofilament 200 [Sigma-Aldrich, Burlington, MA, United States (1:200)], rabbit anti-Myosin VIIa [Proteus BioSciences, Ramona, CA, United States (1:200)], goat anti-Sox10 [Santa Cruz Biotechnology, Santa Cruz, CA, United States (1:80)], rabbit anti-Kir4.1 [Alomone, Jerusalem, Israel (1:200)] and mouse anti-Na,K-ATPase (α1) [DSHB, Iowa City, IA, United States (1:50)]. Secondary antibodies were biotinylated and binding was detected by labeling with fluorescein (FITC)-conjugated avidin D, Texas Red-conjugated avidin D (Vector Labs, Burlingame, CA, United States) or Alexa Fluor Dyes (ThermoFisher Scientific, Waltham, MA, United States). Nuclei were counterstained with propidium iodide (PI).

Sections were examined with a Zeiss Axio Observer (Carl Zeiss Inc., Jena, Germany), and the captured images were processed using Image-Pro Plus software (Media Cybernetics, MD). Quantitative analysis was done using AxioVision 4.8.2 (Carl Zeiss, Inc., Jena, Germany) software. Regions of interest (ROI) in the auditory nerve section were defined using the software outline tool. Apical, middle, and basal portions of the cochleae were examined (27). Within each measured area (or ROI), total cell numbers were determined by counting PI counterstained cell nuclei using the software measurement tool. For quantitative analysis of strial intermediate cell function, cochlear frozen sections of fB^−/−^ and wildtype mice were immunostained using Kir4.1 antibody, a marker of strial intermediate cells. After identifying the ROI, a grayscale was applied to the image using the Automatic Measurement Feature in Axiovision 4.8.2. This function allows for the accurate identification of fluorescent light and provides a means to quantitatively determine regions of Kir4.1 immunoreactivity. Subsequently, fluorescence density measurements were determined by calculating immunostaining intensity and dividing by the ROI area. Three to 7 slides were randomly selected from each cochlea and used for data collection. For the count of IBA1^+^ macrophages, only macrophages having at least one component with a diameter of more than 5 μm or visible nuclei were counted. The method of statistical analysis is also described below.

### 2.4. Confocal microscopy

Sections were examined on a Zeiss LSM5 Pascal (Carl Zeiss Inc.) confocal microscope, a Zeiss LSM 880 NLO, or Leica TCS SP5 (Leica Microsystems, Allendale, NJ, United States) confocal microscope. FITC and Texas Red signals were detected by excitation with the 488 nm and 543 nm lines, respectively. Images were scanned at image scales of 225.0 μm (x) × 225.0 μm (y), 144.72 μm (x) × 144.72 (y), and 450.0 μm (x) × 450.0 μm (y). Captured images were processed using Zen 2012 Blue acquisition software (Carl Zeiss Inc.), Leica Application Suite X software (Version 3.0.2.16120), and Adobe Photoshop CS6 (Adobe Systems Inc., San Jose, CA, United States).

### 2.5. Transmission electron microscopy

Young adult and postnatal WT and fB^−/−^ mice were used for electron microscopy analysis. These mice included 2 young adult WTs, 2 young adult fB^−/−^ mice, 2 P14 WTs, 2 P14 fB^−/−^ mice, 1 P16 WT, and 1 fB^−/−^ mouse. Mice were anesthetized and perfused via a cardiac catheter with 10 mL of normal saline containing 0.1% sodium nitrite followed by 15 mL of a mixture of 4% paraformaldehyde and 2% glutaraldehyde in 0.1 M phosphate buffer, pH 7.4. Cochleae were removed and perfused through the oval window with approximately 3 mL of the fixative mixture. Inner ears were dissected and immersed in the fixative agent overnight at 4°C. Cochleae were decalcified by immersion in 40 mL of 120 mM solution of EDTA, pH 7.0, with gentle stirring at room temperature for 2–3 days with daily changes of the EDTA solution. Cochlear tissues were postfixed with 1% osmium tetroxide-1.5% ferrocyanide for 2 h in the dark, then dehydrated and embedded in Epon LX 112 resin. Ultrathin sections (70 nm thick) were stained with uranyl acetate and lead citrate and examined and imaged using a JEOL JEM-1010 transmission electron microscope (JEOL USA, Inc., Peabody, MA, United States). The middle portions of the cochleas were assessed for the structural integrity of the sensory hair cell, and cells in the auditory nerve and stria vascularis. Pathologies of the auditory nerve and stria vascularis were characterized using criteria outlined in previously published studies (25, 28, 29). The observer was not blinded to the genotype while assessing micrographs across each strain. For light microscopy of toluidine-blue stained sections, Epon-embedded sections with 1 μm thickness were stained with 10% toluidine blue for 1 min, rinsed in H_2_O, and allowed to air-dry overnight before imaging.

### 2.6. RNA isolation and sequencing

To examine the expression of complement-related genes in the developing cochlea, RNA profiling was performed on P3, P7, P14, and P21 CBA/CaJ mice. To examine the consequences of fB deficiency on RNA expression, young adult WT (C57BL/6 J) and fB^−/−^ mice were used. Auditory nerves of mice were isolated by microdissection of the modiolus from structures of the cochlear bulla, cochlear lateral wall, and sensory epithelium. Tissue collected from both cochleae of each mouse was pooled to make an individual sample. Total RNA was purified by miRNeasy Mini Kit (Qiagen Inc., Germantown, MD, United States) per manufacturer instructions. The quality of total RNA preparations was assessed by Agilent 2,100 Bioanalyzer; low-quality samples (RIN <7) were excluded from the study. Sequencing was done on three biological replicates (*n* = 3) of each sample type at the Medical University of South Carolina Genomics Resource. Libraries were prepared using standard Illumina protocols and the TruSeq RNA Library Prep Kit (Illumina Inc., San Diego, CA, United States). Samples were sequenced on an Illumina HiSeq 2,500. Sequencing data were analyzed using Partek^®^ Flow^®^ software (Partek Inc., St. Louis, MO, United States). Reads were aligned to mouse genome assembly mm10 by TopHat2 and quantified to an annotation model (Partek E/M) using mm10 with the following criteria: (a) strict paired-end compatibility, (b) junction reads to match introns required, (c) minimum read overlap with feature at 100% of reading length, and (d) minimum of 10 reads. Comparative analysis was done with DESeq2 (30). Differential expression for complement factor B deficiency was defined as an adjusted *p*-value (FDR step up) <0.05 and an absolute fold change >1.5. Biological process enrichment analysis was conducted with ToppGene (31). For the focused analysis of complement gene expression during AN development, a list of complement genes was compiled based on literature review and gene ontology information; differential expression of these genes was defined as adjusted *p*-value (FDR step up) <0.05 for at least one pairwise relationship. Raw sequencing data (fastq files) and comparison results are archived in NCBI Gene Expression Omnibus (Complement Factor B study, accession GSE182417; Developmental study, GSE133823).

### 2.7. Statistical analysis

For analyses apart from RNAseq, quantitative data are expressed as mean ± SEM, unless otherwise specified. For ABR thresholds, latencies, and wave I amplitudes, linear mixed-effects regression (LMER) models were fit and analyzed using R language [version 4.0.5; R Core Team (2021) and *lme4* package (32)]. LMER is a non–parametric statistical approach that can test hypothesis-driven relationships between predictor and outcome variables while accounting for random variation within and between groups. The interaction models for thresholds included fixed effects of genotype, age, and stimulus frequency, with a random effect of the mouse identification number. Interaction models for latencies and amplitudes included fixed effects of genotype, age, and sound level, with a random effect of the mouse identification number. Type III ANOVA [*car* package; (33)] was run on the LMER models. Degrees of freedom were estimated using the Satterthwaite approximation and rounded to the nearest whole number. *Post-hoc* pairwise comparisons were conducted using the *emmeans* package (34) to obtain estimated marginal means and contrasts, adjusting for multiple comparisons using the Tukey method. Neuron and macrophage count data and Kir4.1 immunofluorescence measure data were analyzed using GraphPad Prism 7 software (GraphPad Software, Inc. La Jolla, CA) for Windows and significance was determined by Mann-Whitney U test. For all statistical tests, a *p*-value of ≤ 0.05 was considered significant. The sample size is indicated in each figure or figure legend.

## 3. Results

### 3.1. Characterization of complement molecules and factor B in the mouse cochlea

**Figure 1:**
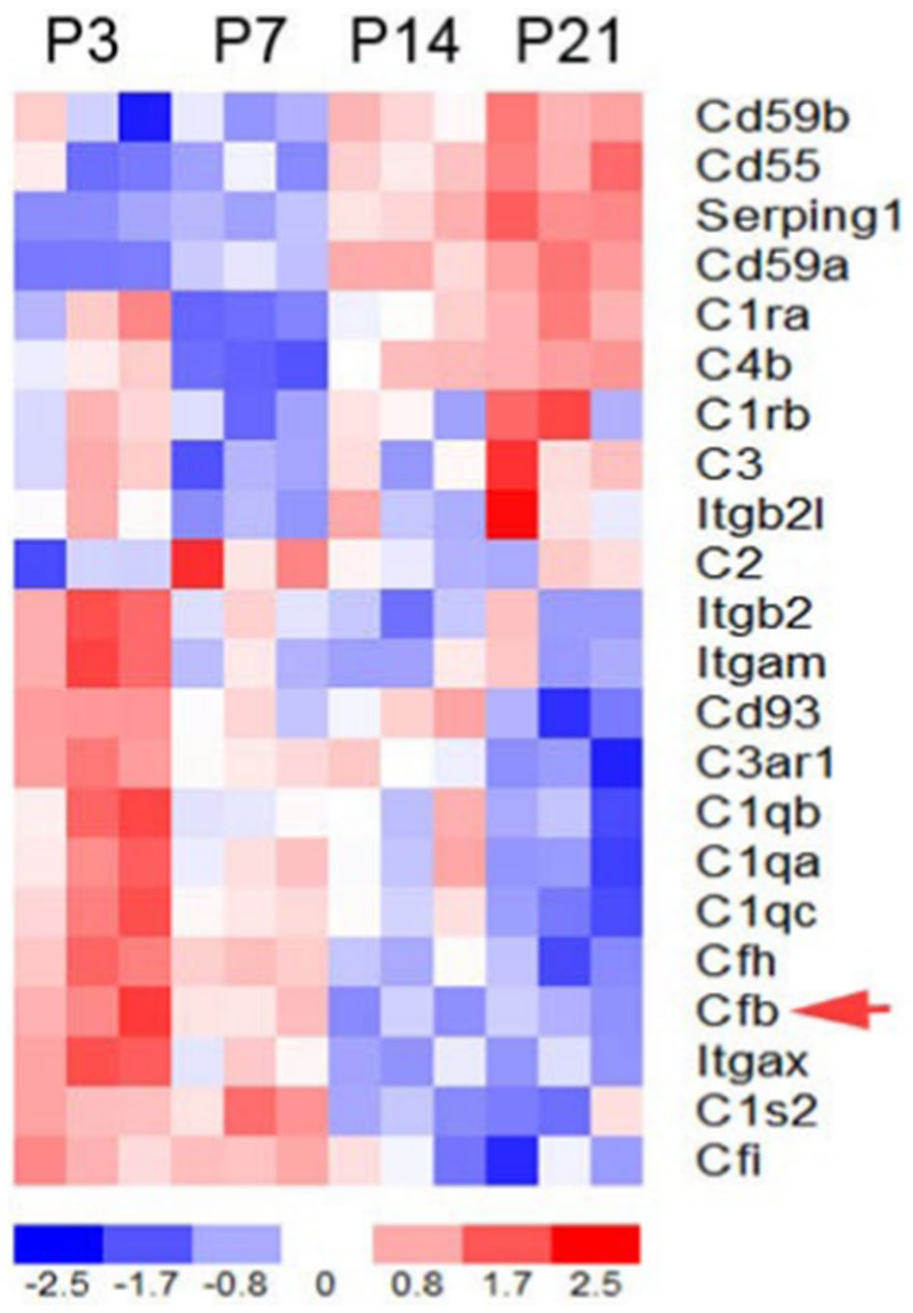
Complement gene expression in the postnatal developing auditory nerve of CBA/CaJ mice. Expression profiles of key complement genes in postnatal auditory nerves at P3, P7, P14, and P21. The red arrow indicates fB expression. Colorimetric scaling (Z-standardization) for the heatmap is shown at the bottom.

To determine the expression patterns of complement genes in the developing mouse inner ear, we performed RNA sequencing analysis on auditory nerve samples of CBA/CaJ mice aged P3, P7, P14, and P21 (Figure 1). Many key complement genes were differentially expressed in the early postnatal ear (Supplementary Table S1). Clustering analysis of these genes identified two groups of complement-related genes that differed in expression pattern during cochlear development: a group of genes with initially low expression at P0 and a noticeable increase in expression between P7 and P14, and the second group of genes whose expression steadily decreased from P3 to P21, a critical period marked by myelination, hearing onset, and auditory function enhancement. We found fB to be among the cluster with decreasing expression (red arrow).

### 3.2. Progressive hearing impairment in mice with factor B deficiency

**Figure 2:**
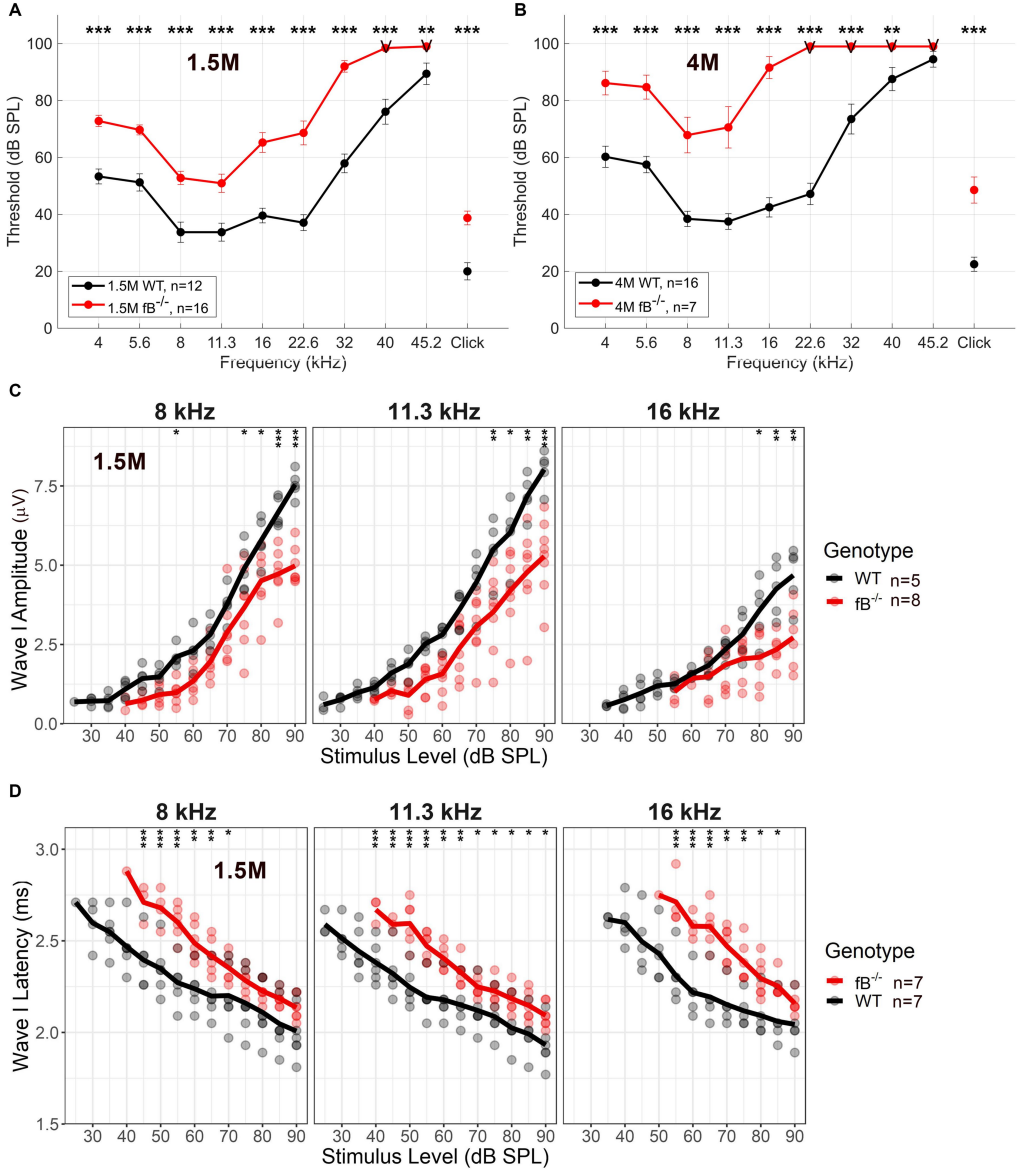
Auditory function in fB deficient mice. Auditory Brainstem Response (ABR) was measured in adult WT and fB^−/−^ mice. **(A,B)** ABR thresholds in 1.5-month **(A)**, and 4-month-old **(B)** fB^−/−^ mice (red line) and age-matched WT mice (black line). LMER model of the ABR threshold indicated significant effects between WT and fB^−/−^ mice (see Results section). *Post-hoc* pairwise comparisons were conducted to compare genotypes for each frequency at each age, and significant differences between genotypes are denoted in **(A,B)** with asterisks (*p < 0.05, **p < 0.01, ***p < 0.001). **(C)** Average and individual wave I amplitudes for 1.5-month fB^−/−^ and WT mice (randomized subset taken from mice tested in **A**) in response to 8, 11.3, and 16 kHz stimulus. LMER models of ABR wave I amplitude showed a significant main effect of genotype and stimulus sound pressure level at 8 kHz, 11.3 kHz, and 16 kHz (see Results section). *Post-hoc* pairwise comparisons were conducted to compare amplitudes across genotypes for each sound level, and significant differences between genotypes are denoted with asterisks (*p < 0.05, **p < 0.01, ***p < 0.001). **(D)** Average and individual wave I latencies for 1.5-month fB^−/−^ and WT mice (randomized subset taken from mice tested in **A**) in response to 8, 11.3, and 16 kHz stimulus. LMER models of ABR wave I latency revealed a significant main effect of genotype and stimulus sound pressure level (see Results section) for 8, 11.3, and 16 kHz. *Post-hoc* pairwise comparisons were conducted to compare latencies across genotypes for each sound level, and significant differences between genotypes are denoted with asterisks (*p < 0.05, **p < 0.01, ***p < 0.001). Error bars in **(A,B)** represent SEM. “V” in **(A,B)** indicates that most fB^−/−^ mice showed no response at the examined frequencies.

To investigate the impact of fB on auditory function, we utilized a previously generated mouse model of targeted fB deficiency (fB ^−/−^ mice) (20, 35). We analyzed auditory brainstem responses (ABR) of wave I thresholds at 1.5 months and 4 months in age-matched WT and fB ^−/−^ mice. At 1.5 months, fB^−/−^ mice demonstrated significantly increased ABR thresholds at all frequencies tested compared to the WT mice (Figure 2A). Additionally, 4-month-old fB^−/−^ mice continued to demonstrate significantly higher ABR thresholds than the WT mice (Figure 2B). ANOVA results using the LMER model of the ABR threshold indicated significant effects of genotype [*F*(1,116) = 4.1162, *p* = 0.045], age [*F*(2,670) = 13.052, *p* < 0.001], and frequency [*F*(9,651) = 26.385, *p* < 0.001], two-way interactions of genotype and age [*F*(2,203) = 3.537, *p* = 0.031], genotype and frequency [*F*(9,621) = 4.674, *p* < 0.001], and age and frequency [*F*(18,651) = 7.028, *p* < 0.001], and a three-way interaction of genotype, age, and frequency [*F*(18,651) = 3.702, *p* < 0.001]. The significant effect of age on the ABR threshold LMER model, indicated a progressive hearing loss in adult fB^−/−^ mice.

ABR supra-threshold function measures revealed decreased wave I amplitudes and delayed wave I latencies at 8, 11.3 and 16 kHz [which represents the apical-middle portion of the cochlea (36); Figures 2C,D]. LMER models of ABR wave I amplitude showed a significant main effect of genotype for the 8 kHz [*F*(1,18) = 31.155, *p* < 0.001], 11.3 kHz [*F*(1,11) = 93.786, *p* < 0.001], and 16 kHz [*F*(1,10) = 12.996, *p* = 0.004] stimulus, in addition to interactions of genotype and stimulus sound pressure level for 8 kHz [*F*(10,101) = 3.362, *p* < 0.001], 11.3 kHz [*F*(10,113) = 3.780, *p* < 0.001] and 16 kHz [*F*(7,80) = 5.25, *p* < 0.001]. Further, LMER models of ABR wave I latency revealed a significant main effect of genotype for the 11.3 kHz [*F*(1,20) = 7.127, *p* = 0.015] tone pips, in addition to interactions of genotype and stimulus sound pressure level for the 8 kHz [*F*(10,113) = 2.818, *p* = 0.004], 11.3 kHz [*F*(10,117) = 2.943, *p* = 0.002], and 16 kHz [*F*(8,80) = 3.464, *p* = 0.002] stimulus. Together these ABR wave I suprathreshold data suggest that pathophysiological alterations occur in the peripheral auditory nerve of adult fB^−/−^ mice.

### 3.3. Gene expression alterations in the auditory nerve of factor B deficient mice

**Figure 3:**
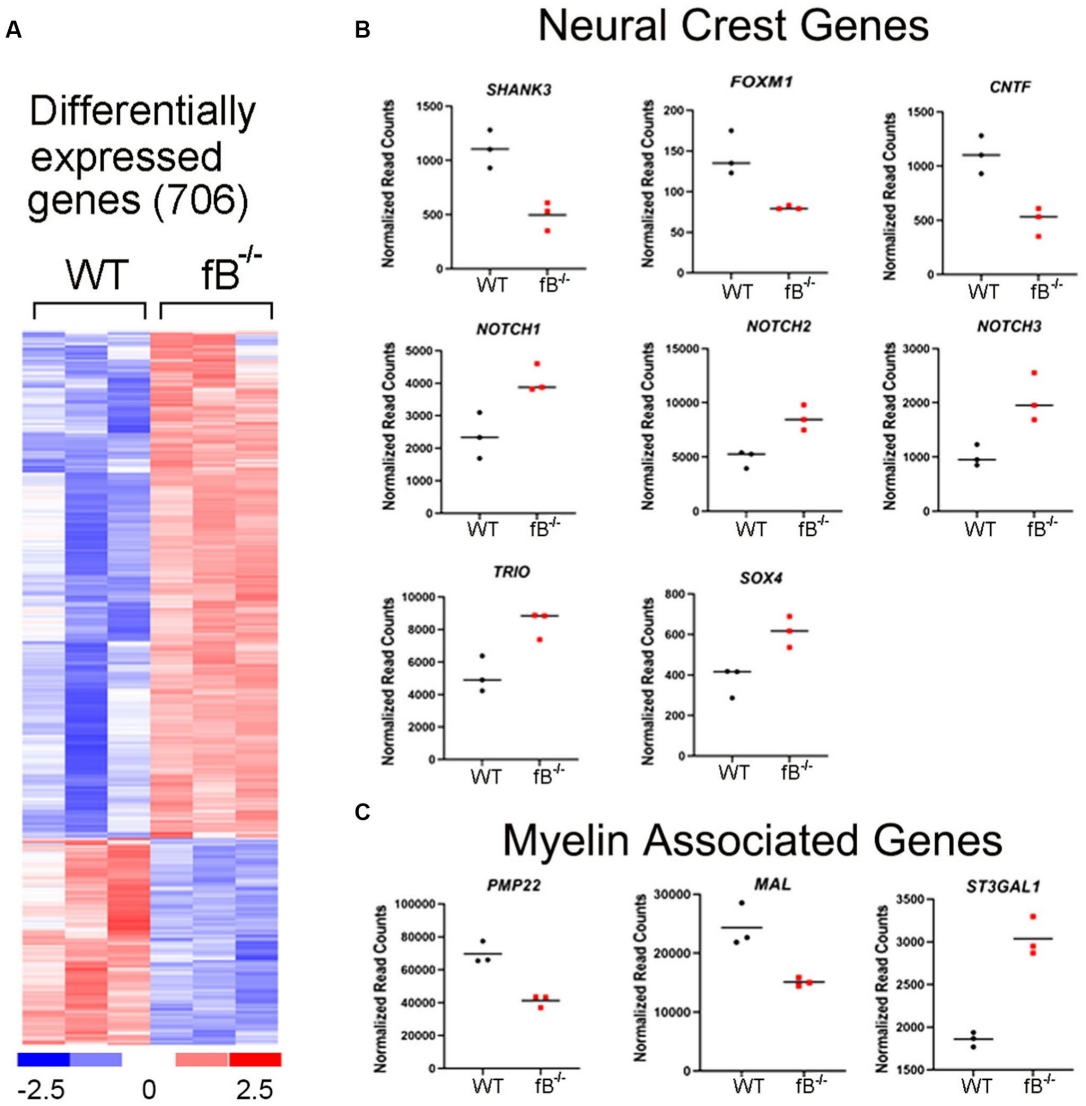
Gene expression changes in the auditory nerve of fB^−/−^ mice. **(A)** Heatmap representation of genes differentially expressed between fB^−/−^ and WT mice. Comparison of auditory nerve tissue from young adult (aged 1.5–2.5 months) fB^−/−^ and WT control animals found that 706 genes were significantly differentially expressed (*p*-adj <0.05 and absolute fold change >1.5), with 501 being upregulated and 205 being downregulated. **(B,C)** Read count representation of neural crest genes (*Shank3*, *Foxm1*, *Cntf*, *Notch1*, *Notch 2*, *Notch 3*, *Trio*, and *Sox4*) and myelin-associated genes (*Pmp22*, *Mal*, and *St3gal1*) identified as differentially expressed.

The significant differences in hearing sensitivity and auditory nerve function in fB^−/−^ mice compared to WT mice suggest that pathological changes may have occurred within the auditory nerve of these animals. To further investigate this finding at the molecular level, we performed RNA-sequencing (RNAseq) on the auditory nerves of young adult fB^−/−^ mice to assess gene expression changes that may relate to or contribute to hearing impairment. As shown in Figure 3A, RNAseq analyses identified over 700 genes that were differentially expressed when comparing auditory nerves from adult fB^−/−^ mice to WT mice (*p*-adjusted <0.05, absolute fold change >1.5; Supplementary Table S2). Analysis of biological processes enriched among the differentially expressed genes identified processes associated with cell adhesion, extracellular structure organization, neurogenesis, and generation of neurons (Table 1).

**Table 1:** Gene function enrichment analysis for biological process changes in the auditory nerve of fB^−/−^ mice.

Biological process (top 25 GO names)	*q*-value Bonferroni
Extracellular structure organization	3.91E-11
External encapsulating structure organization	4.67E-11
Cell adhesion	3.45E-09
Biological adhesion	4.52E-09
Neurogenesis	6.35E-07
Anatomical structure formation involved in morphogenesis	2.26E-06
Cell morphogenesis	3.52E-06
Generation of neurons	8.65E-06
Heart development	1.26E-05
Cellular component morphogenesis	1.32E-05
Cell morphogenesis involved in differentiation	2.96E-05
Cytoskeleton organization	3.07E-05
Supramolecular fiber organization	5.32E-05
Embryo development	9.96E-05
Neuron differentiation	1.10E-04
Circulatory system development	2.14E-04
Behavior	2.36E-04
Cognition	8.85E-04
Central nervous system development	1.03E-03
Sensory organ development	1.20E-03
Cell morphogenesis involved in neuron differentiation	1.31E-03
Embryonic morphogenesis	1.84E-03
Head development	2.19E-03
Neuron projection development	2.25E-03
Regulation of cell differentiation	2.44E-03

Glia cells, including satellite and Schwan cells in the peripheral auditory nerve, are of neural crest origin (37, 38). Interestingly, several genes related to neural crest cell differentiation and myelin function were among those differentially expressed in fB^−/−^ mice (Figures 3B,C). Among these genes, *Notch1*, *Notch2*, *Notch3*, *Trio*, *Cntf*, and *Shank3* are active during neural crest cell migration and differentiation (39–41). *Foxm1* is a gene needed for the renewal of neural progenitor and proliferation of glial cells (42, 43). *Sox4* is a regulator of Schwann cell differentiation (44). Lymphocyte protein (MAL) is an integral membrane protein and plays a role in maintaining myelination (45). Peripheral myelin protein 22 (PMP22) is a key protein that forms the compact myelin of the peripheral nervous system (46). Collectively the differential expression of these genes suggests that fB activity is important for the development and function of neural crest cells and myelination in the auditory nerve.

### 3.4. Pathology of glial cells in the auditory nerve of factor B deficient mice

**Figure 4:**
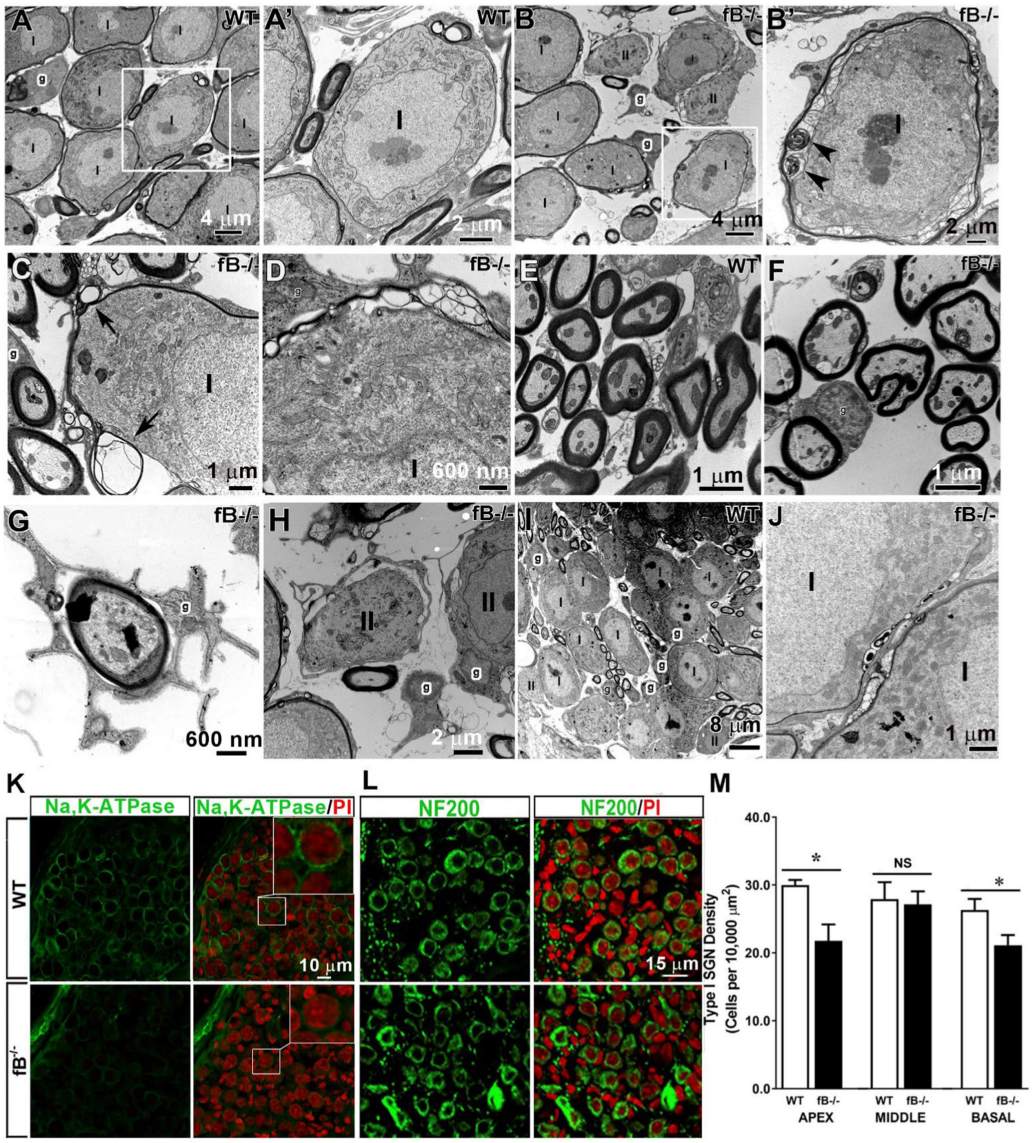
Pathophysiological changes of glial cells in the auditory nerve of fB knockout mice. **(A–H)** Electron micrographs of Rosenthal’s canal in adult WT **(A,A’,C,I)** and fB^−/−^ **(B,B′,D–H,J)** mice. **(A’)** is the high magnification of the enclosed areas in the WT **(A)** auditory nerve revealing tight associations between the myelin of satellite glial cells (g) and type I **(I)** SGNs. **(B′)** is the high magnification of the enclosed area in **(B)** showing abnormal swirling of myelin sheaths around type I SGNs (black arrowheads) of fB^−/−^ mice. Similar changes in myelination of SGNs were seen in type I SGNs of the other young adult fB^−/−^ mice **(C,D)**. Arrowheads point out myelin whorls in the myelination surrounding SGNs in **(C)**. **(E–G)** Electron micrographs of axons in the osseous spiral lamina (OSL) of WT **(E)** and fB^−/−^ **(F,G)** mice. The majority of myelinating glial cells around the axon appear relatively normal in the fB knockout mice **(F)**. Enlarged space between the glial cells and the axon of the auditory nerve in an fB^−/−^ mouse **(G)**. **(H)** Pathological changes were also seen in the non-myelinating glial cells around type II (II) SGNs. **(I,J)** Similar myelin pathological changes were also seen in the postnatal fB^−/−^ mice (P14) **(J)**, although the development of SGNs shows no abnormalities **(I)**. **(K)** Confocal images showing Na,K-ATPase immunoreactivity in satellite glial cells surrounding SGNs in young adult WT (the top panel) and fB^−/−^ (bottom panel) mice. Confocal images of both WT and fB^−/−^ mice were obtained using the same microscope acquisition settings. Images in the top-right of the right panel are enlarged images of boxed areas in the center. A reduced Na,K-ATPase immunoreactivity is seen between WT and fB^−/−^ mouse samples. Images were taken from the basal portion of the mouse cochleas. **(L,M)** Neuron densities are significantly reduced in the apical and basal portions but not in the middle portion in fB^−/−^ mouse cochleae. Confocal images of NF200^+^ neurons in the middle turn of young adult WT (the top panel) and fB^−/−^ (the bottom panel) mice. Propidium iodide (PI) was used to counterstain cell nuclei. Scale bar = 20 μm. Quantification of SGNs in WT and fB^−/−^ mice revealed significant decreases of SGNs in the apical and basal portions of the auditory nerve and no significant loss of SGNs **(M)**. Data bars represent mean density; error bars represent SEM; Data were analyzed by Mann-Whitney U test with statistical significance defined as a *p value of ≤ 0.05 (see detailed information in the Result section).

In the auditory nerve of young adult WT mice, type I spiral ganglion neuron (SGN) cell bodies and axons of the auditory nerve were myelinated with satellite cells and Schwann cells, respectively (Figures 4A,A’,E). Myelin cell pathologies were present in the auditory nerves of fB knockout mice, including abnormal swirling of myelin sheaths, presence of myelin blebs, and enlarged spaces between satellite cells and the ensheathed SGNs (Figures 4B–D), while satellite cells closely flanked the SGNs in WT auditory nerves. Although the majority of auditory nerve axons showed relatively normal myelination (Figures 4E,F), some Schwann cell abnormalities around the axons were seen, including atypically wide spacing between the Schwann cell cytoplasm and the adjacent axon in fB^−/−^ mice (Figure 4G). Abnormal non-myelinating satellite cells were also present around some of the type II SGNs (Figure 4H). In addition, abnormal development of myelination was seen in the auditory nerve of P14 fB^−/−^ knockout mice (Figure 4J) compared to the aged-matched WT animals (Figure 4I).

To assess the impact of fB deficiency on glial cell function in the auditory nerve, we examined the expression of Na,K-ATPase using an antibody that can identify the α1 isoform of Na,K-ATPase protein. Both α1 and α2 isoforms are expressed in the glial cells of many nervous tissues (47, 48). In particular, Na,K-ATPase α1 was recently reported to express exclusively on satellite glial cells of the human auditory nerve (49). Immunostaining revealed a reduced immunoreactivity of Na,K-ATPase on the satellite glia around SGNs in the auditory nerves of fB^−/−^ mice (Figure 4K), in agreement with pathological alterations of myelin around SGNs identified by EM observation (Figures 4B–D). Together, these results suggest functional declines of auditory nerve activity in fB^−/−^ mice.

To visualize the SGNs, we immunostained auditory nerves with NF200, a marker of sensory neurons. Staining and quantification of SGNs revealed a significant reduction in the number of SGNs in fB^−/−^ mice compared to WT mice in the apical and basal (but not middle) portions of the cochlea (Figures 4L,M) (Mann-Whitney U test; *p* = 0.03 for apex; *p* = 0.47 for middle; *p* = 0.04 for the base). The mean ± SEM (cells per 10,000 μm^2^) of NF200^+^ SGN densities are 29.98 ± 0.7 (*n* = 4) and 21.97 ± 2.41 (*n* = 3) in the apex, 27.92 ± 2.48 (*n* = 7) and 27.15 ± 1.91 (*n* = 6) in the middle, and 26.32 ± 1.62 (*n* = 5) and 21.13 ± 1.50 (*n* = 4) in the base for WT and fB^−/−^ mice, respectively. This suggests that fB deficiency has a negative impact on the SGNs in the apical and basal portions of the cochlea.

**Figure 5:**
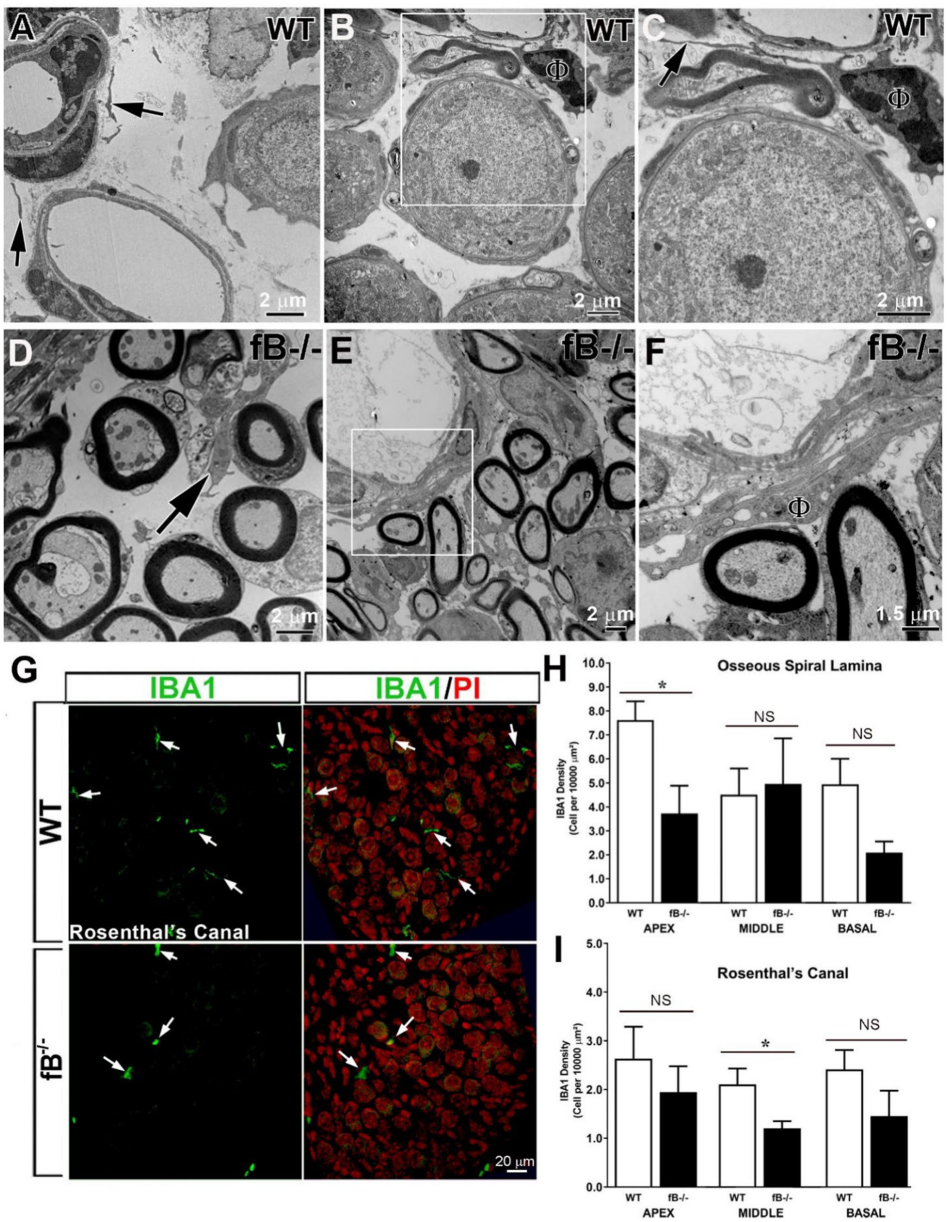
Complement fB deficiency does not significantly alter macrophage numbers in the adult auditory nerve. **(A–F)** Electron micrographs of macrophages (black arrows) in the auditory nerve of young adult WTs **(A–C)**, young adult fB^−/−^ **(C)** and P16 fB^−/−^ **(B,C)** mice. **(C)** is a magnification of the boxed area in **(B)** showing a macrophage (ϕ) around a blood vessel with a long and thin cellular process (arrow). An arrow in **(D)** identifies a macrophage around the axons of the auditory nerve. **(F)** is a magnification of the boxed area in **(E)** showing an activated macrophage (ϕ) around a blood vessel. **(G)** Confocal images of IBA1^+^ macrophages in young adult WT (top panels) and fB^−/−^ (bottom panels) mice. The images were taken within Rosenthal’s canal (RC) of the middle portions of the mouse cochleas. Nuclei were counterstained with PI. **(H–I)** Quantification of IBA1^+^ macrophages revealed no significant changes in macrophage numbers in most cochlear areas except in the apical portion within the osseous spiral lamina (OSL) and middle portion within RC in fB^−/−^ mice. Data bars represent mean; error bars represent SEM. For the auditory nerve in the OSL, *n* = 7 and 6, *n* = 6 and 5, and *n* = 6 and 4 cochleas for WT and fB^−/−^ mice in the apex, middle and base, respectively; For the auditory nerve within the RC, *n* = 5 and 4, *n* = 6 and 5, and *n* = 7 and 5 cochleas for WT and fB^−/−^ mice in the apex, middle and base, respectively. Analysis was done with the Mann-Whitney U test; statistical significance was defined as a *p value of ≤0.05.

Immune cells, such as macrophages, are another key element of the innate immune system, and a major source of complement protein production [see review (50)]. Recent studies have demonstrated that macrophage phenotype is impacted by the presence of complement factors (51–54). We hypothesized that macrophage activity is reactive to changes in complement signaling and therefore, fB deficiency may lead to a reduction of macrophage activity in the auditory nerve. Using ultrastructural analysis, we identified macrophages in several areas of the cochlea in WT and fB^−/−^ mice. In the auditory nerves of the WT mice, it is relatively difficult to identify macrophages. The cellular processes of these macrophage-like cells are often thinner with limited vacuoles (Figures 5A–C). Surprisingly, macrophages in fB^−/−^ mice contained increased lysosomes, abundant rough endoplasmic reticulum, and increased phagoliposomes and vacuoles (24, 25, 28, 55), characteristics that suggest these macrophages were in an activated state. Activated macrophages were seen throughout the auditory nerves of fB^−/−^ mice, particularly in regions around the auditory nerve microvasculature (Figures 5D–F), opposing our original hypothesis. Immunodetection of IBA1, a calcium-binding protein expressed in the cytoplasm of microglia/macrophages, also showed that macrophages were present in the cochlea of fB knockout mice (Figure 5G). Here, we found that macrophages demonstrated a range of morphologies between fb^−/−^ and WT mice, suggesting a diverse range of macrophage functions and phenotypes. In agreement with the EM observations, IBA1^+^ cells were identified with fewer long cellular processes in the auditory nerves of fB knockout mice (Figure 5G), suggesting an activated state of macrophages in fB^−/−^ mice. However, quantitative analysis of IBA1^+^ cells detected no significant differences in the numbers of macrophages between WT and fB^−/−^ mice in most of the cochlear areas, except a reduction was seen in the apical cochlear portion within the osseous spiral lamina (OSL) and the middle cochlea portion within the Rosenthal’s canal (RC) in the fB^−/−^ mouse auditory nerves (Figures 5H,I) (*p* values for the auditory nerve in the OSL are 0.021, 0.331, and 0.086 for the apex, middle and base, respectively; *p* values for the auditory nerve within the RC are 0.278, 0.026, and 0.134 for the apex, middle and base, respectively). These findings suggest that fB deficiency induces macrophage activation and phenotypic alterations, yet has a limited impact on macrophage numbers in the cochlea highlighting the complex dynamic between macrophages and complement signaling.

### 3.5. Histopathology of the stria vascularis in factor B deficient mice

**Figure 6:**
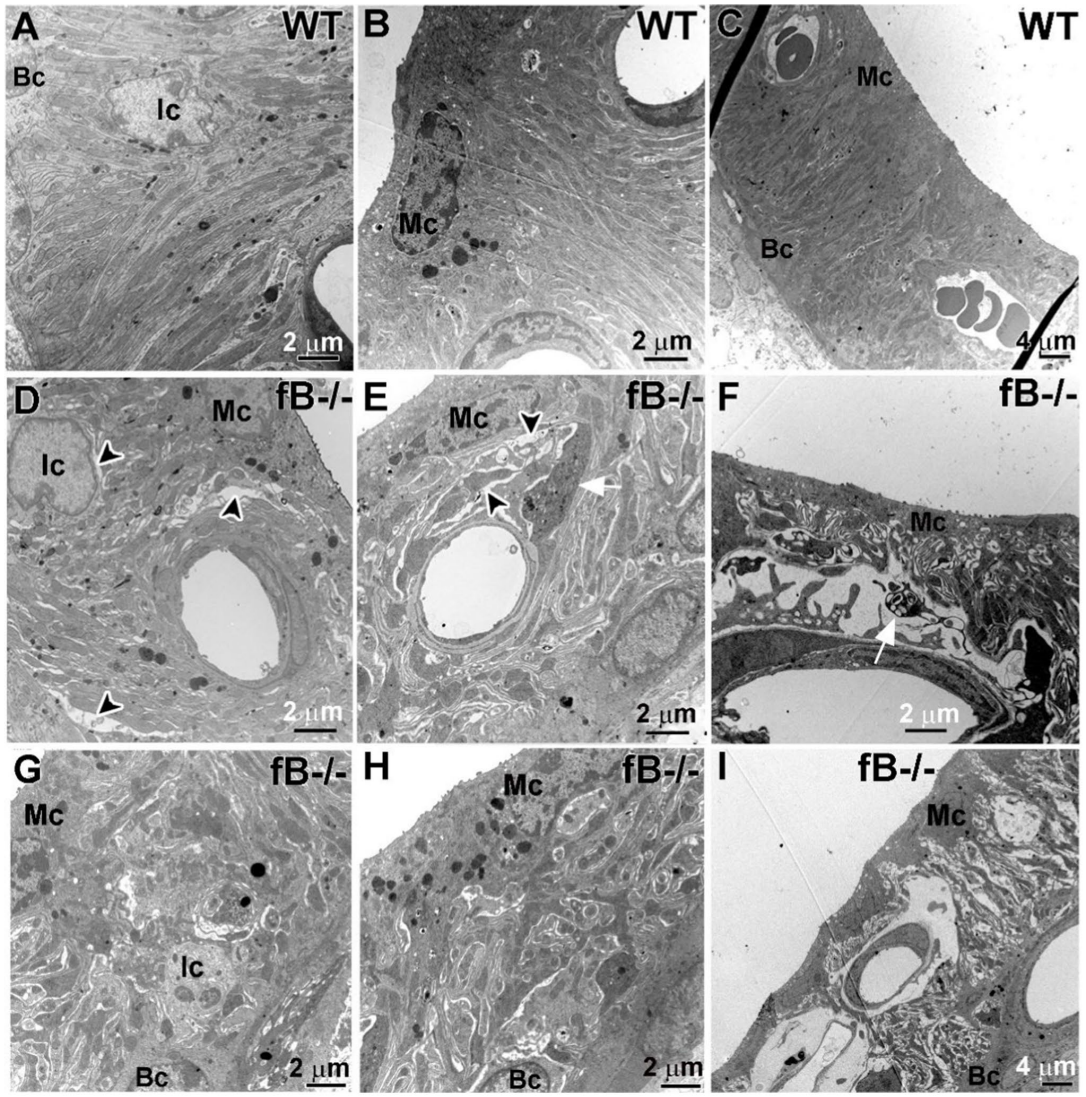
fB deficiency results in cochlear stria vascularis pathologies. **(A–F)** Electron micrographs of the stria vascularis in the cochlear lateral wall of WT **(A–C)** and fB^−/−^ **(D–I)** mice. In young adult fB^−/−^ mice **(D,E,G,H)**, the integrity of the interdigitation among intermediate cells (Ic), marginal cells (Mc) and basal cells (Bc) is disrupted or lost (black arrowheads), while their age-matched WT controls have well-organized interdigitation in the stria vascularis **(A–C)**. **(F,I)** Substantial pathological alterations were seen in the strial vascularis of postnatal fB^−/−^ mice (P16 mouse in **F**; P14 mouse in **I**). A white arrow shows macrophage-like processes within the space between the intermediate cells and marginal cells.

Given that perivascular-resident macrophage-like melanocytes reside in the stria vascularis (56) and that strial intermediate cells are also neural crest-derived cells (57), we examined if fB deficiency leads to pathological alterations in the stria vascularis of the cochlear lateral wall. In the stria vascularis of WT mice, the mitochondria-enriched cytoplasmic processes of strial intermediate cells interdigitate tightly with the more electron-dense processes of marginal cells and the fibrous process of the basal cells (Figures 6A–C). However, in fB^−/−^ mice, cells in the stria vascularis appeared to be separated by a larger space, particularly in postnatal animals (Figures 6D–I). Additionally, processes extending from activated macrophages were rarely apparent in the stria vascularis of the WT mice, but these cells were readily detected in the stria vascularis of fB^−/−^ mice (Figure 6E). These macrophage-like cells were identified by special features of activated macrophage including densely packed cytoplasm filled with multiple lysosomes, electron-dense lipid bodies and phagoliposomes as described in our previous studies (24). These strial pathologies and activated macrophages were also seen in postnatal fB^−/−^ mice (Figure 6F). For both young adult and postnatal mice, the well-organized interdigitation among marginal cells, intermediate cells, and basal cells in the stria vascularis was mostly disrupted or lost in fB^−/−^ mice (Figures 6D–I).

**Figure 7:**
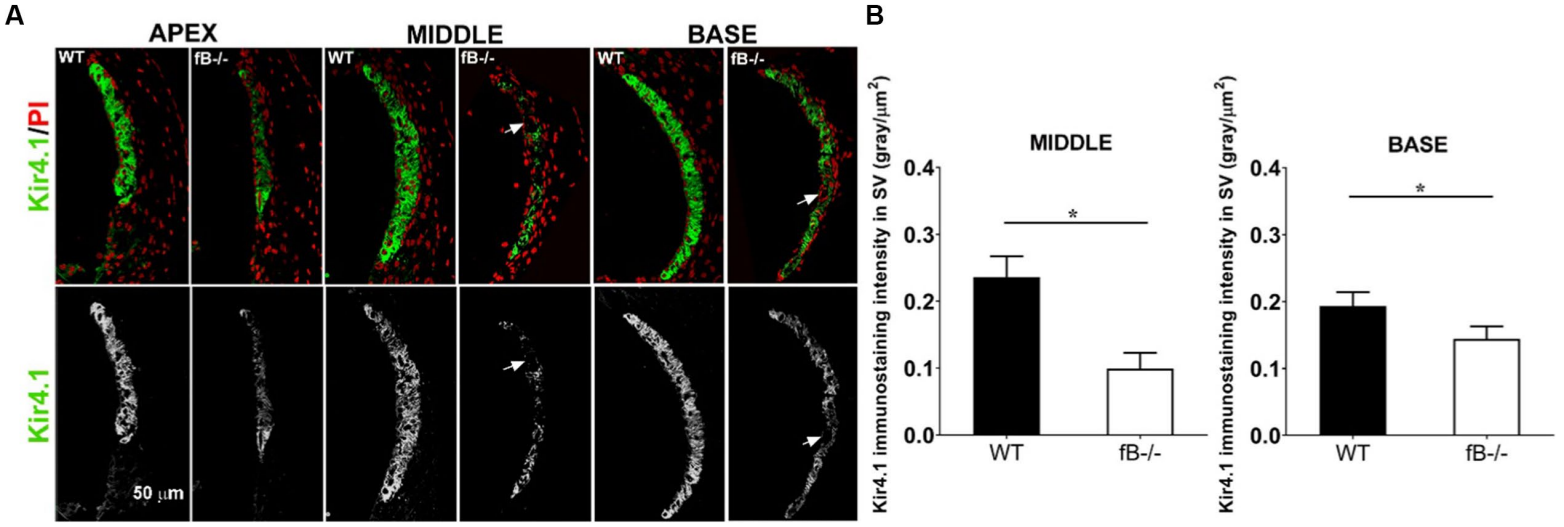
Kir4.1 immunoreactivity reduction in fB^−/−^ mice. **(A)** Confocal representations of Kir4.1 staining in the cochlear lateral wall of WT and fB^−/−^ mice reveal diminished expression in fB^−/−^ mouse cochleae. Propidium iodide (PI) was used to counterstain cell nuclei. **(B)** A significantly reduced Kir4.1 immunoreactivity was seen in the middle and basal stria vascularis of the fB^−/−^ mice. Data bars represent the mean; error bars represent SEM. *n* = 6 and 5 cochleas for the middle turn in WT and fB^−/−^ group, respectively; *n* = 7 and 5 cochleas for the basal turn WT and fB^−/−^ group, respectively. Analysis was done with the Mann-Whitney U test; statistical significance was defined as a *p value of ≤ 0.05.

Next, we examined the changes of the potassium channel proteins Kir4.1 and Na,k-ATPase in the stria vascularis in fB^−/−^ mice. Kir4.1 and several isoforms of Na,k-ATPase including α1 have been used to evaluate the functional state of the strial intermediate cells and marginal cells, respectively (49, 58–60). Both proteins are essential for generating the endocochlear potential, in the cochlear lateral wall (59, 61, 62). We found that Kir4.1 immunoreactivity was substantially diminished in fB^−/−^ mice (Figure 7A). Kir4.1 immunoreactivity was measured by the fluorescence density of Kir4.1 immunostaining (Mann-Whitney U test; *p* = 0.015 for both the middle and base). There were also additional instances where the integrity of the stria vascularis was compromised, evidenced by gaps in Kir4.1 immunostaining, suggesting a total loss of Kir4.1 immunoreactivity in these strial areas of fB^−/−^ mice (Figure 7A arrows). Focusing on the basal turn of the cochlea, an area where we saw a severe loss of marginal cell-intermediate cell interdigitation, quantitative analysis revealed significantly diminished Kir4.1 immunoreactivity in fB^−/−^ mice (Figure 7B). However, limited changes in Na,K-ATPase immunoreactivity in the marginal cells were seen in the stria vascularis (Supplementary Figure S1). Together with the electron microscopy data shown in Figure 6, these immunostaining data suggest that fB activity plays a role in maintaining the structural and functional integrity of the strial intermediate cells, a neural crest derived cell in the cochlear lateral wall.

### 3.6. Cochlear hair cells remain in factor B deficient mice

**Figure 8:**
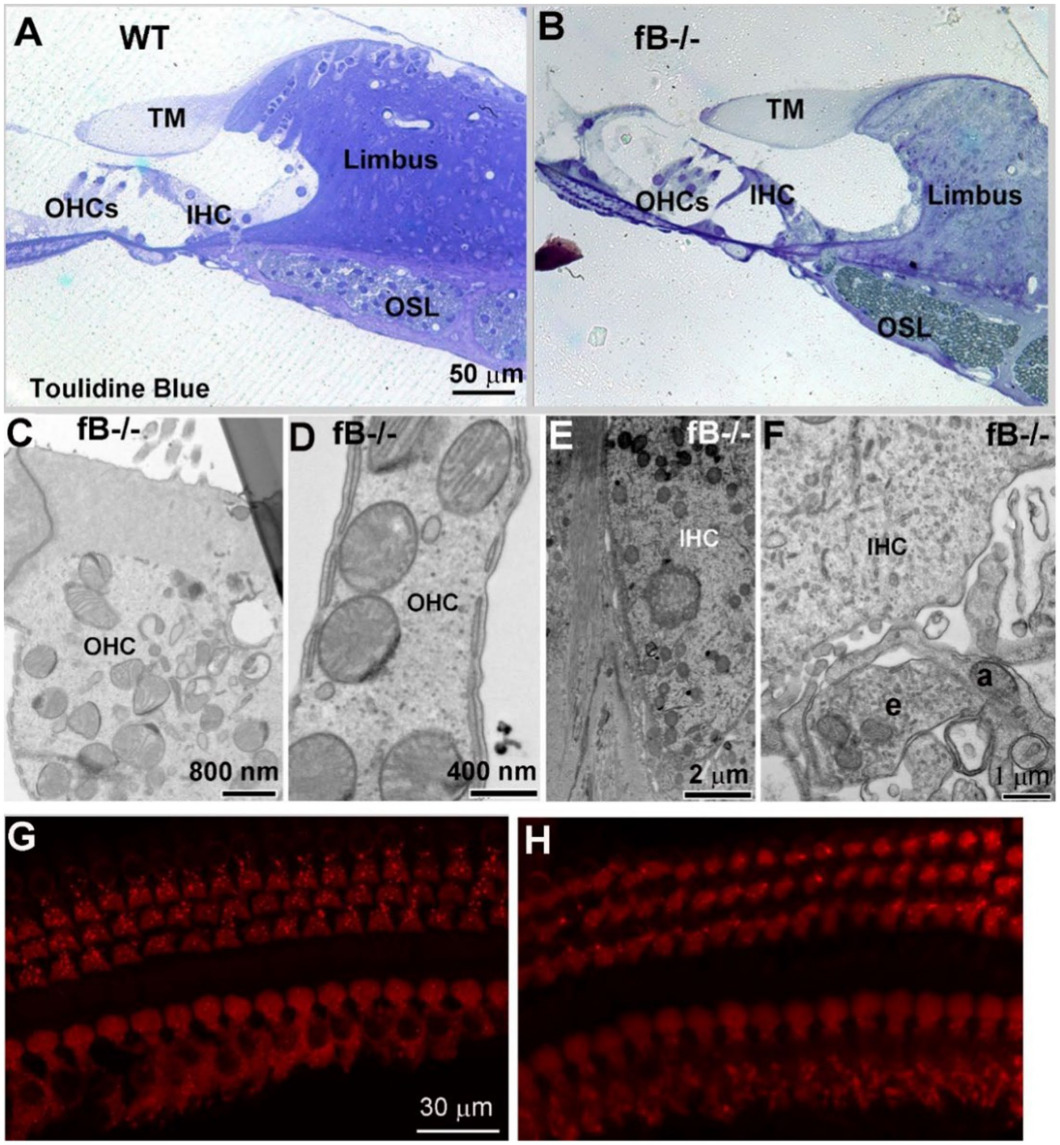
The organ of Corti histology is comparable in fB^−/−^ and WT mice. **(A,B)** Light microscopy of toluidine blue-stained 1-month WT and fB^−/−^ cochlear samples. Representative images of the organ of Corti of WT **(A)** and fB^−/−^ **(B)** mice. TM, tectorial membrane; OSL, osseous spiral lamina; OHC, outer hair cell; IHC, inner hair cell. Images in **(A,B)** were representative images taken from the middle portion of mouse cochleas. Two mice per group were observed. **(C–F)** Normal ultra-structures of OHCs and IHCs in young adult fB^−/−^ cochleas. An efferent (e) terminal connects with an afferent terminal (a) underneath of an inner hair cell. **(G,H)** Confocal microscopy of MyosinVIIa^+^ hair cells in WT **(G)** and fB^−/−^ **(H)** cochleae. Hair cells are well preserved in both mouse strains.

Given the hearing impairment of fB^−/−^ mice, we evaluated organ of Corti structures in both WT and fB^−/−^ mice. Toluidine blue staining of cochlear sections (Figures 8A,B) and ultrastructural examination (Figures 8C–F) revealed that there was minimal pathological alteration in sensory hair cells of young adult fB^−/−^ mice. To validate this result, we analyzed the immunoreactivity of the hair cell marker MyosinVIIA. We did not find a significant loss in inner or outer hair cells of fB^−/−^ mice (Figures 8G,H). Together, these results suggest that fB deficiency has limited impact on sensory hair cell morphology.

## 4. Discussion

The complement system is widely expressed in central and peripheral nerves. Complement signaling has been associated with several neurological disorders, including traumatic brain injury, age-related macular degeneration, and Alzheimer’s disease (63–68). While most studies focus on the impact of the classical complement pathway, the role of alternative complement signaling in neuronal dysfunction and disorder remains to be explored. Using a well-established mouse model of fB deficiency (20), our study demonstrated that alternative complement signaling is required for the development and maintenance of auditory function. Mice deficient in fB signaling displayed elevated ABR thresholds, decreased ABR wave I amplitudes, and delayed ABR wave I latencies. Additionally, we found that adult fB knockout mice displayed multiple pathologies of the peripheral auditory system, including abnormalities in the auditory nerve and stria vascularis of the cochlear lateral wall. Our study is the first to reveal that proper functioning of the alternative complement pathway is required for the development and maintenance of two key non-sensory cochlear cell types, glial cells, and intermediate cells, which are derived from the neural crest.

The alternative complement pathway acts as a feedback mechanism, via amplifying complement signaling as fB binds to hydrolyzed C3b proteins. Alternative complement signaling has been linked to increased inflammation in the brain after traumatic brain injury (69, 70) and retinal tissue during age-related macular degeneration (71). In addition, fB^−/−^ mice that have undergone cerebral ischemia–reperfusion injury demonstrate reduced neurological pathologies and display protection from demyelination (22). Further, inhibition of the alternative complement pathway, via administration of neutralizing antibodies or genetic deletion, results in decreased neuroinflammation and decreased neurodegeneration in traumatic brain injury and multiple sclerosis (69, 72, 73), suggesting that depleting alternative complement signaling can be protective under pathological conditions. Other studies have shown that fB deletion can be protective and generate positive impacts (74, 75). For example, rats with genetically depleted fB exhibit increased metabolic and cardiovascular function in spontaneous hypertensive rodents. Collectively, these observations suggest that the relationship between fB signaling and disease exacerbation or amelioration may be dependent on disease models and the severity of pathological conditions. However, the effect of fB signaling deficiency in a non-pathological or developmental context is less well-studied. Here we observed that fB deficiency elicited hearing loss at all ages tested, from young adult through old adult stages. Our results support the view that fB signaling is required for normal hearing onset and maintenance of adult auditory function in the non-pathological condition.

Using RNA-seq, we found that several biological processes were significantly impacted by fB deficiency in the auditory nerve, including extracellular structure and organization, neurogenesis, cognition and behavior, central nervous system development, and generation of neurons. When analyzing differentially expressed genes in fB knockout mice, we found several genes that were associated with neural crest cell development and myelination. Interestingly, we observed a significant loss in the number of spiral ganglion neurons in fB knockout compared to control mice in both the apical and basal portions of the cochleas. We also found obvious pathologies in the myelin sheath of the auditory nerve and abnormalities in the stria vascularis of the cochlear lateral wall. Recent studies found that complement genes contribute to neural crest cell migration during embryonic development (76, 77). Given that auditory glia and cochlear melanocytes (such as intermediate cells of the stria vascularis) are derived from neural crest cells (28, 49, 78), fB may be important for neural crest cell function. Further, glial cell-specific pathologies may be the underlying cause for delayed ABR wave I latencies seen in fB^−/−^ mice (Figure 2). Additionally, auditory glial cells support, nourish, protect, and influence the function of spiral ganglion neurons (79–81). Given the important supportive role that glial cells exert on neighboring neurons, the presence of pathology among glial cells in the auditory nerve in fB^−/−^ mice could be the causative factor of the decreased spiral ganglion neuron numbers we identified in these fB knockout mice.

Our results also revealed the indirect influence of fB signaling on the maintenance of cochlear ion homeostasis. When analyzing Kir4.1 in the cochlear lateral wall of fB^−/−^ mice, we found that expression of this protein was significantly reduced, when compared to WT mice. Kir4.1 is critical for the rectifying of K^+^ by intermediate cells in the cochlear lateral wall for proper generation and maintenance of the endocochlear potential (82, 83). Also, Kir4.1 channels on the surface of glial cells play a critical role in siphoning extracellular K^+^ released by neurons during activity (84, 85). Mice lacking Kir4.1 signaling demonstrate profound hearing loss due to a reduced endocochlear potential, and a diminished ability of glial cells to expel SGN-released K^+^ ions (82, 85). Although not investigated in this study, future studies should focus on determining if the pathological alterations that we identified in the stria vascularis of the cochlear lateral wall results in reduced endocochlear potential in fB^−/−^ mice.

Since there were no apparent pathologies in the structures of the organ of Corti, including the inner and outer hair cells, it is likely auditory dysfunction in fB^−/−^ mice is largely due to the pathologies in the auditory nerve and cochlear lateral wall. Although we did not examine hair cell functional alterations in this study (e.g., changes in distortion product otoacoustic emissions), our data revealed increased ABR thresholds and impaired suprathreshold functioning, indicating dysfunction of the peripheral auditory nerve in young adult mice. A previous study found limited cochlear pathological alterations in animal models deficient in the classical complement molecule *C1qa* (86). Hair cell specific *complement C1q like 1* (*C1ql1*) deletion leads to no changes in hearing sensitivity (15), however, a significant hearing loss together with auditory nerve pathology has been reported in the global *C1ql1* deficiency model (16). The alternative complement pathway is responsible for ~80% of complement activity (87). Thus, our finding of hearing impairment in fB^−/−^ mice highlights the importance of comprehensive investigation into alternative complement signaling. Further studies should be aimed at exploring (1) molecular regulation of alternative complement signaling in the development and function of neural crest derived cells in the cochlea, and (2) if alternative complement signaling contributes to hair cell innervation and the maturation of pre- and postsynaptic connections in the auditory nerve.

Additionally, deficiency in fB has been linked to the suppression of immune responses. For instance, previous literature has shown that mice deficient in both fB and C2 of the alternative pathway were at a higher risk of contracting renal fungal infections (74, 88). These mice also demonstrated a delayed opsonophagocytic effect and subsequently displayed higher levels of infection in the kidneys (88). Also, fB knockout mice displayed lower airway responsiveness to the toxic agent methacholine and demonstrated remarkably less airway inflammation (89).

Interestingly, we found limited changes in the macrophage numbers between fB^−/−^ and WT mice. However, we did find macrophages in activated states in fB-deficient mice, although we did not further investigate phenotypic changes in cochlear macrophages. Studies show that macrophages respond to molecular signaling changes in their microenvironment (90–92). Considering the regulatory role that complement signaling exerts on macrophages, and the previously established role of cochlear macrophages in auditory nerve development (24, 93, 94), cochlear injury repair (95–98), and stria vascularis function, (56, 99, 100), future studies should be aimed at examining potential macrophage-specific alterations related to complement signaling, such as the regulation of pro-inflammatory and anti-inflammatory macrophage activation states. Additionally, given the important role that macrophages play in the inflammaging of the mouse cochlea (4, 101), it will be important to further investigate the pathological alterations in aged fB^−/−^ mice.

Here we report the important role of the complement pathway in hearing function and the preservation of the cochlear structure. We found that complement proteins, including factors related to the alternative signaling pathway, are expressed in the developing and adult mouse cochlea. Mice deficient in fB signaling exhibited progressive hearing impairment, however, these animals do not appear to have robust hair cell abnormalities. While SGNs in the auditory nerves of fB^−/−^ mice appeared unaltered, severe pathology was discovered in the ultrastructure of satellite/Schwann cells in the auditory nerve, and abnormal architecture in the stria vascularis of the cochlear lateral wall was identified. Collectively, these results indicate that alternative complement signaling is important for peripheral auditory system function. Additional studies should elucidate the source of complement signaling in the cochlea and the mechanism by which the alternative complement pathway exerts its influence.

## Data availability statement

The datasets presented in this study can be found in online repositories. The names of the repository/repositories and accession number(s) can be found in the article/Supplementary material.

## Ethics statement

The animal study was reviewed and approved by the Institutional Animal Care and Use Committee of MUSC.

## Author contributions

LB, JB, CA, and HL contributed to the conception and design of the study. LB, SJ, and HL performed the research. LB, JB, SJ, JR, TJ, and HL analyzed the data. CA provided the animal model used in the study. LB and HL wrote the manuscript. All authors contributed to the article and approved the submitted version.

## Funding

This work was supported by National Institutes of Health Grants F31DC014435 (LB), T32DC014435 (LB, TJ, JR), K18 DC018517 (HL), R56DC012058-06 (HL), R01DC012058 (HL), P50DC000422 (HL), SFARI Pilot Award #649452 (HL), P30GM103342 (JB), P20GM103499 (JB), UL1 TR00145, P30 CA138313, and S10 OD018113 from Cell and Molecular Imaging Shared Resource and Hollings Cancer Center, C06 RR014516 from the Extramural Research Facilities Program of the National Center for Research Resources, and the Medical University of South Carolina Office of the Vice President for Research.

## Conflict of interest

The authors declare that the research was conducted in the absence of any commercial or financial relationships that could be construed as a potential conflict of interest.

## Publisher’s note

All claims expressed in this article are solely those of the authors and do not necessarily represent those of their affiliated organizations, or those of the publisher, the editors and the reviewers. Any product that may be evaluated in this article, or claim that may be made by its manufacturer, is not guaranteed or endorsed by the publisher.
