# Supplementary material for: Complement factor B is essential for the proper function of the peripheral auditory system
Source: Front Neurol. 2023 Jul 25;14:1214408. doi: 10.3389/fneur.2023.1214408 (PMC10408708; doi:10.3389/fneur.2023.1214408)
Supplement: Supplementary file 3 [file Table_3.docx]

Supplementary Table 3. Antibodies used in the study

| **Primary Antibody** | **Host** | **Company** | **Catalog No.** | **Concentration** |
| --- | --- | --- | --- | --- |
| Anti-IBA1 | Polyclonal rabbit IgG | Wako Chemicals (Richmond,VA) | 019-19741 | 1:200 |
| Anti-NF200 | Monoclonal mouse IgG | Sigma-Aldrich (St. Louis, MO) | N0142 | 1:200 |
| Anti-Kir4.1 | Polyclonal rabbit IgG | Alomone Labs (Jerusalem, Israel) | PC035AN0802 | 1:200 |
| Anti-MyosinVIIa | Polyclonal Goat IgG | Proteus BioSciences (Ramona, CA) | 25-6790 | 1:200 |
| **Secondary Antibody** | **Host** | **Company** | **Catalog No.** | **Concentration** |
| Biotinylated Anti-Goat IgG | Horse | Vector Laboratories (Burlingame, CA) | BA-9500 | 1:100 |
| Biotinylated Anti-Rabbit IgG | Horse | Vector Laboratories (Burlingame, CA) | BA-1100 | 1:100 |
| Biotinylated Anti-Mouse IgG | Goat | Vector Laboratories (Burlingame, CA) | BA-9200 | 1:100 |
| **Anti-Biotin Dyes** | **Host** | **Company** | **Catalog No.** | **Concentration** |
| Fluorescein Avidin DCS | N/A | Vector Laboratories (Burlingame, CA) | A-2011 | 1:100 |
| Texas Red® Avidin D | N/A | Vector Laboratories (Burlingame, CA) | A-2006 | 1:100 |
