# Supplementary figures and images for: Complement factor B is essential for the proper function of the peripheral auditory system
Source: Front Neurol. 2023 Jul 25;14:1214408. doi: 10.3389/fneur.2023.1214408 (PMC10408708; doi:10.3389/fneur.2023.1214408)

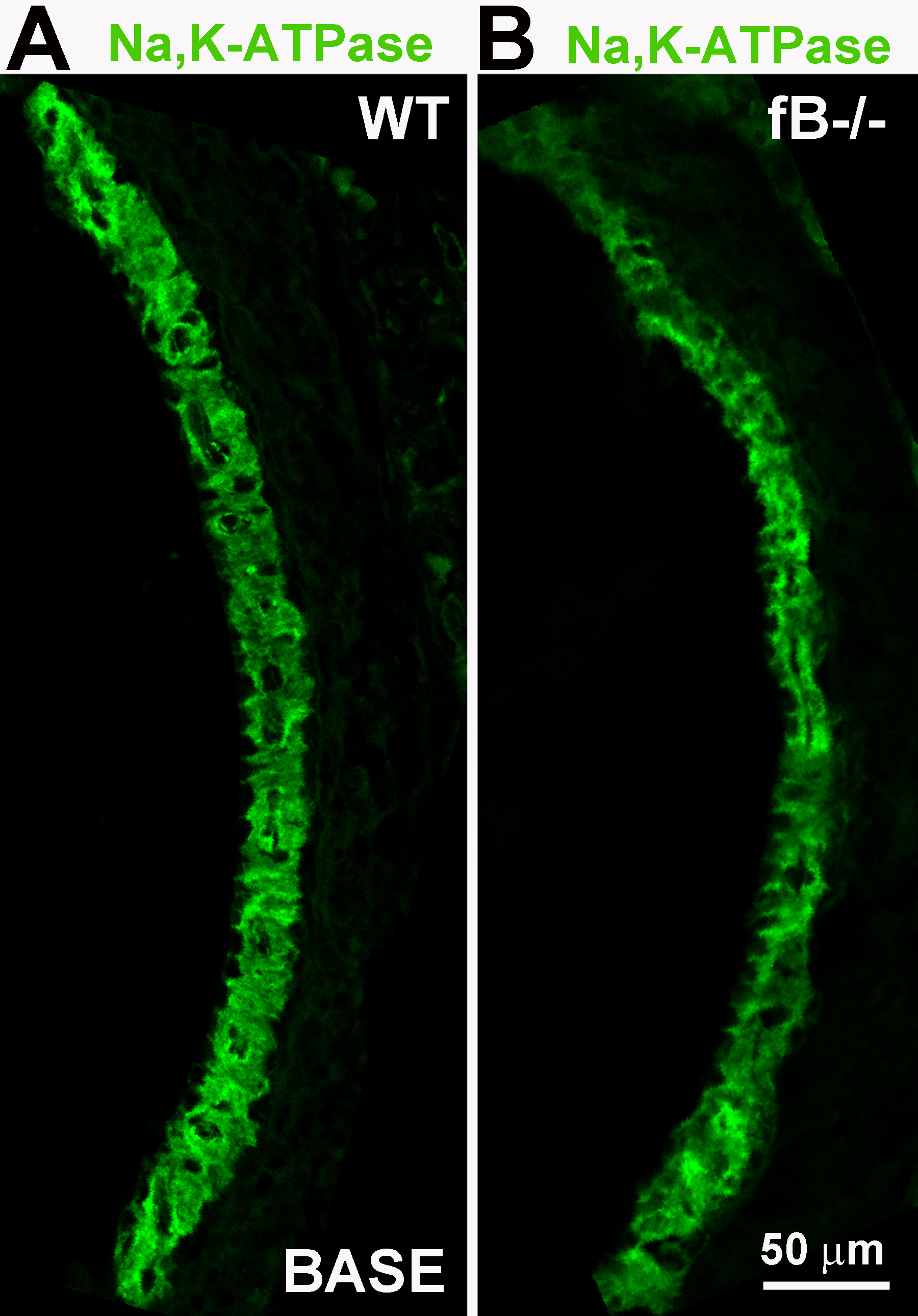

Supplement: Supplementary file 4 [file Image_1.jpg]
